# Radial probe endobronchial ultrasound‐guided transbronchial lung biopsy for the diagnosis of cavitary peripheral pulmonary lesions

**DOI:** 10.1111/1759-7714.13980

**Published:** 2021-05-04

**Authors:** Kyung Soo Hong, Jong Geol Jang, June Hong Ahn

**Affiliations:** ^1^ Division of Pulmonology and Allergy, Department of Internal Medicine, College of Medicine Yeungnam University and Respiratory Center, Yeungnam University Medical Center Daegu South Korea

**Keywords:** bronchoscopy, cavity, peripheral, ultrasonography

## Abstract

**Background:**

Cavitary peripheral pulmonary lesions (PPLs) are often diagnosed via transthoracic needle biopsy. However, today, radial probe endobronchial ultrasound (RP‐EBUS) is widely used to diagnose PPLs. The efficacy and safety of RP‐EBUS‐guided transbronchial lung biopsy (RP‐EBUS‐TBLB) used to diagnose cavitary PPLs remain poorly known. We investigated the utility of RP‐EBUS‐TBLB using a guide sheath (GS) without fluoroscopy to diagnose PPLs.

**Methods:**

Of 743 RP‐EBUS procedures conducted to diagnose PPLs performed at our institution from January 2019 to October 2020, we analyzed 77 cavitary PPLs. TBLB was performed using RP‐EBUS with a GS without fluoroscopy. The diagnostic accuracy and complications were assessed. All lung lesions with a definitive diagnosis were included in analyses.

**Results:**

The overall diagnostic accuracy was 85.7% (66/77). Of malignant lesions (*n* = 34), 29 (85.3%) were diagnosed successfully. Of benign lesions (*n* = 43), 37 (86.0%) were diagnosed successfully. In multivariate analyses, a thicker cavity wall (≥10 mm, odds ratio [OR] 14.22, 95% confidence interval [CI] 2.58–78.35, *p* = 0.002) and EBUS imaging with the probe within the lesion (OR 12.02, 95% CI 1.91–75.53, *p* = 0.008) independently affected diagnostic success. The likelihood of success increased with increasing thickness of the cavity wall (*p* < 0.001, test for trend). The specimens obtained for molecular confirmation of malignancy were satisfactory. There were four cases of infection (5.2%) and three cases of pneumothorax (3.9%).

**Conclusions:**

RP‐EBUS‐TBLB of cavitary PPLs affords high diagnostic accuracy with acceptable complication rates.

## Graphical abstract

(A) Computed tomography (CT) scan of a 58‐year‐old man with a 16‐mm diameter spiculated cavitary nodule in the left upper lobe. (B) Radial probe endobronchial ultrasound (RP‐EBUS) revealed the EBUS image within the lesion; biopsy was performed using a brush and forceps. Histology revealed an adenocarcinoma. (C) CT scan of a 59‐year‐old man with a 32‐mm diameter cavitary mass in the left upper lobe. (D) RP‐EBUS yielded an EBUS image within the lesion, and biopsy was performed. Histology and microbiology specimens revealed pulmonary tuberculosis.

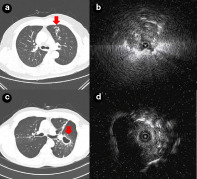

## INTRODUCTION

A pulmonary cavity is a gas‐filled space within a zone of pulmonary consolidation, a mass, or a nodule. A cavity may reflect necrosis, cystic dilation of a lung region in patients with benign diseases and necrosis, internal cyst formation, or internal desquamation of tumor cells in patients with malignant diseases.^1^ The differential diagnosis is broad and includes various infections, autoimmune conditions, and primary and metastatic malignancies.^2^ Radiological findings of benign and malignant cavitary peripheral pulmonary lesions (PPLs) often overlap; tissue analyses are required when diagnosing a PPL.

Transthoracic needle biopsy (TTNB) has commonly been used to diagnose PPLs. The diagnostic accuracy is close to 90%,^3^ but a meta‐analysis found that the overall complication rates after core biopsy and fine needle aspiration (FNA) are 38.8% and 24.0%, respectively.^4^ The same is true after diagnosis of cavitary PPLs. TTNB is associated with a diagnostic rate of over 80%, but the complication rate is elevated by more than 20%.^5^, ^6^ Recently, radial probe endobronchial ultrasound (RP‐EBUS) has become widely used to diagnose PPLs. The pooled sensitivity of RP‐EBUS‐guided transbronchial lung biopsy (RP‐EBUS‐TBLB) is 0.72 and the safety profile is excellent.^7^

Diagnosis of the cavitary PPLs is different from that of the noncavitary PPLs, which means that the cavitary wall should be targeted for biopsy. However, its diagnostic efficacy and the safety of RP‐EBUS for diagnosing cavitary PPLs remain poorly known. In this study, we analyzed the clinical utility of RP‐EBUS in terms of diagnosing cavitary PPLs.

## MATERIALS AND METHODS

### Study design and subjects

**FIGURE 1:**
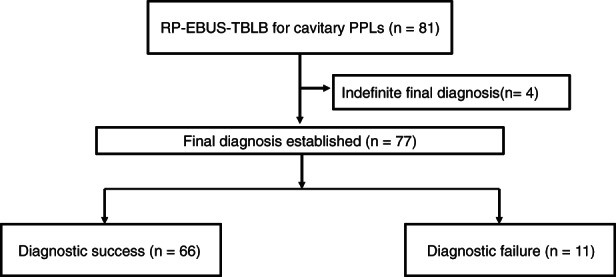
Study flowchart. PPLs, peripheral pulmonary lesions; RP‐EBUS‐TBLB, radial probe endobronchial ultrasound‐guided transbronchial lung biopsy

Of 743 RP‐EBUS procedures performed to evaluate PPLs at Yeungnam University Hospital from January 2019 to October 2020, we performed a retrospective observational study on 81 consecutive patients who underwent RP‐EBUS‐TBLB to explore cavitary PPLs. Of the biopsied lesions, 77 were definitively diagnosed according to biopsy results (*n* = 66), clinical course (*n* = 1), sputum microbiology (*n* = 5), liver biopsy (*n* = 1), EBUS‐transbronchial needle aspiration (*n* = 3), and surgical resection (*n* = 1). Four cases with indefinite diagnoses (neither benign nor malignant) were excluded from the diagnostic accuracy analyses. A study flowchart is presented in Figure 1.

### CT preparation and biopsy

All patients underwent thin‐section chest computer tomography (CT) (0.75‐mm slice thickness at intervals of 0.75 mm; Somatom Definition AS 64‐slice CT system, Siemens Healthcare) prior to biopsy. Three pulmonologists (Kyung Soo Hong, Jong Geol Jang, and June Hong Ahn) reviewed the images before the procedure and defined the pathways to be used when accessing cavitary PPLs. The bronchus sign of CT was defined as a bronchus leading to the target lesion. The distance from the lesion to the pleura was the shortest distance evident in the axial CT scan.^8^ The cavitary wall thickness was the greatest thickness in the region of the biopsy site apparent in the axial image.

All bronchoscopy procedures were performed by one of the three pulmonologists (above). A 4‐mm diameter bronchoscope (BF P260F, Olympus) was used to attain the target lesions. After the lesions were revealed by RP‐EBUS, biopsy specimens were collected using a brush and biopsy forceps. All RP‐EBUS‐TBLB procedures were performed using a guide sheath (GS) but without X‐ray fluoroscopy.

**FIGURE 2:**
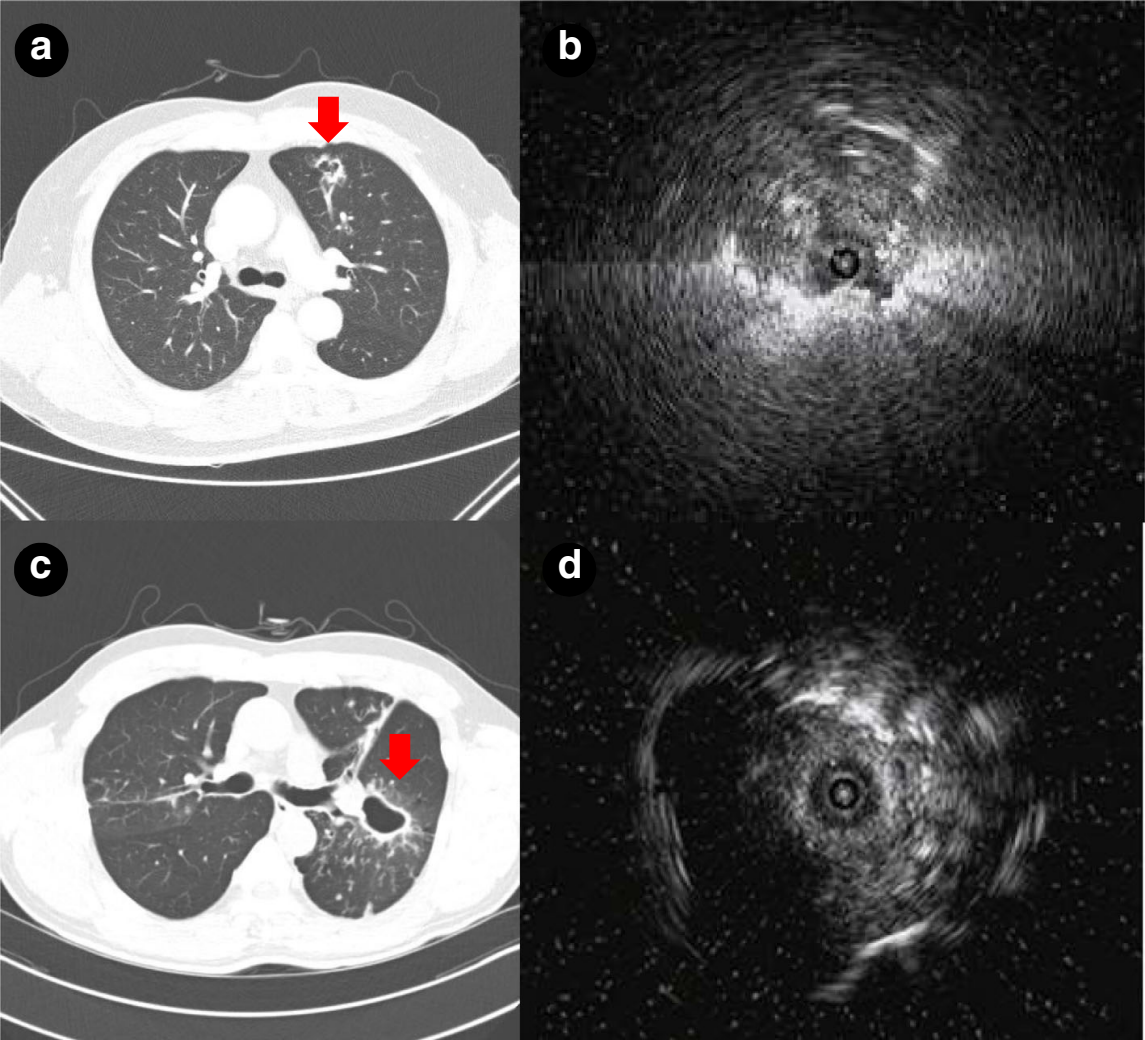
Representative cases. (a) Computed tomography (CT) scan of a 58‐year‐old man with a 16‐mm diameter spiculated cavitary nodule in the left upper lobe. (b) Radial probe endobronchial ultrasound (RP‐EBUS) revealed the EBUS image within the lesion, and biopsy was performed using a brush and forceps. Histology revealed an adenocarcinoma. (c) CT scan of a 59‐year‐old man with a 32‐mm diameter cavitary mass in the left upper lobe. (d) RP‐EBUS yielded an EBUS image within the lesion and biopsy was performed. Histology and microbiology specimens revealed pulmonary tuberculosis

Representative data on RP‐EBUS‐TBLB patients with cavitary PPLs are shown in Figure 2.

### Diagnostic classification

A final diagnosis of malignancy was made based on the definite histological or cytological evidence of malignancy. Benign lung lesion was diagnosed according to the following criteria: identification of definite benign features (at least one of the histology, cytology, or microbiology tests was diagnostic), regression of the lesion with medical treatment, and a stable size for at least 12 months. The lesions initially diagnosed as malignant by RP‐EBUS and finally diagnosed with malignancy were considered true positives. The lesions initially diagnosed as benign by RP‐EBUS and finally diagnosed as benign were considered true negatives. Initial benign results that were finally diagnosed with malignancy were considered false negatives. To explore factors affecting diagnostic accuracy, we divided the study population into two groups: a diagnostic success group (true‐positive and true‐negative results) and a diagnostic failure group (false‐positive and false‐negative results). We calculated the diagnostic accuracy by dividing the number of diagnostic successes by the total number of cases.

### Statistical analyses

Continuous variables were compared using the Student *t* test or the Mann–Whitney U test and are expressed as means ± standard deviations. Categorical variables were compared using the chi‐square or Fisher's exact test and are described as frequencies (percentages). Univariate and multivariate logistic regression analyses were performed to identify factors affecting the diagnostic accuracy. The factors evaluated in multivariate analyses were those significant at the *p* < 0.1 level in univariate analyses. We used the linear‐by‐linear association test to analyze the diagnostic accuracy by cavity wall thickness. We employed the Kruskal–Wallis test to compare the numbers of specimens by cavity wall thickness. In all analyses, a two‐tailed *p* value <0.05 was considered statistically significant. All statistical procedures were performed using SPSS software (version 24.0, IBM).

### Ethics statement

This study was conducted in accordance with the tenets of the Declaration of Helsinki, and the protocol was reviewed and approved by the Institutional Review Board of Yeungnam University Hospital (no. 2020‐09‐025). The requirement for informed patient consent was waived because of the retrospective study design.

## RESULTS

### Baseline characteristics

Of the 743 RP‐EBUS procedures performed for PPL diagnosis in our institution from January 2019 to October 2020, 77 cavitary PPLs were included in this study. The baseline characteristics of the 77 patients are listed in Table 1. The mean age was 68.0 ± 11.9 years and 59 were men (76.6%). Chronic obstructive pulmonary disease (COPD, *n* = 23, 29.9%) was the most common underlying lung disease. The mean predicted FEV1% was 80.9 ± 17.8. Most lung lesions were in the right lower lobe (*n* = 23, 29.9%) and the mean distance from the pleura to the lung lesion was 8.9 ± 10.2 mm. The mean cavity wall thickness was 12.3 ± 5.4 mm. The bronchus sign was positive on CT in 68 patients (88.3%) and EBUS images within the lesion were apparent in 67 (87.0%) patients. The mean procedural time was 17.7 ± 7.1 min.

**TABLE 1:** Baseline characteristics of patients

Characteristic	Total (*n* = 77)
Age, years	68.0 ± 11.9
Male	59 (76.6%)
Underlying lung diseases	
COPD	23 (29.9)
Asthma	5 (6.5)
IPF	3 (3.9)
CPFE	2 (2.6)
Old pulmonary TB	10 (13.0)
Pulmonary function test data	
FEV1_%_	80.9 ± 17.8
FVC%	81.2 ± 17.2
FEV1/FVC ratio	71.8 ± 12.4
Lung lesions	
Location	
RUL	21 (27.3)
RML	1 (1.3)
RLL	23 (29.9)
LUL	19 (24.7)
LLL	13 (16.9)
Distance from pleura (mm)	8.9 ± 10.2
Diameter (mm)	37.1 ± 15.4
Cavity wall thickness (mm)	12.3 ± 5.4
Bronchus sign on CT	
Positive	68 (88.3)
Negative	9 (11.7)
Procedure	
EBUS imaging findings	
Within	67 (87.0)
Adjacent	10 (13.0)
Procedural time (min)	17.7 ± 7.1

*Abbreviations*: COPD, chronic obstructive pulmonary disease; CPFE, combined pulmonary fibrosis and emphysema; CT, computed tomography; EBUS, endobronchial ultrasound; FEV1, forced expiratory volume in 1 s; FVC, forced vital capacity; IPF, idiopathic pulmonary fibrosis; LLL, left lower lobe; LUL, left upper lobe; RLL, right lower lobe; RML, right middle lobe; RUL, right upper lobe; TB, tuberculosis.

### Pathological results and diagnostic performance

The final diagnoses and diagnostic accuracys by the type of sample are shown in Table 2. Of the 77 lung lesions that were ultimately diagnosed, 34 (44.2%) were malignant and 43 (55.8%) were benign. Of the malignant lesions (*n* = 34), 29 (85.3%) were diagnosed successfully via RP‐EBUS‐TBLB. Squamous cell carcinoma of the lung (*n* = 16, 47.1%) was the most common definitive malignant diagnosis. Of the 34 malignant lesions, 21 (61.8%) brushing cytology and 27 (79.5%) histology samples were diagnostic. In terms of brushing cytology, adenocarcinoma (90.9%) was more often diagnosed than squamous cell carcinoma (50.0%). False‐negative lesions (*n* = 5) were diagnosed as malignant by liver biopsy (*n* = 1), surgical resection (*n* = 1), and EBUS transbronchial needle aspiration (*n* = 3).

**TABLE 2:** Final diagnoses and diagnostic accuracys by sample type

Final diagnosis	All (*n* = 77)	Diagnosed via EBUS‐GS, *n* (%)	Diagnosed via brushing cytology, *n* (%)	Diagnosed via histology, *n* (%)	Diagnosed via microbiology, *n* (%)
Malignant	34	29 (85.3)	21 (61.8)	27 (79.4)	0 (0)
Adenocarcinoma	11	11 (100)	10 (90.9)	10 (90.9)	0 (0)
Squamous cell carcinoma	16	14 (87.5)	8 (50.0)	14 (87.5)	0 (0)
NSCLC, NOS	6	4 (66.6)	3 (50.0)	3 (50.0)	0 (0)
Metastatic carcinoma	1	0 (0)	0 (0)	0 (0)	0 (0)
Benign	43	37 (86.0)	1 (2.3)	24 (55.8)	24 (55.8)
Lung abscess	10	10 (100)	0 (0)	10 (100)	0 (0)
Pulmonary tuberculosis	14	12 (85.7)	0 (0)	7 (50.0)	12 (85.7)
Nontuberculous mycobacterial lesion	15	12 (80.0)	0 (0)	4 (26.6)	12 (80.0)
Aspergillosis	1	1 (100)	1 (100)	1 (100)	0 (0)
Inflammation	2	2 (100)	0 (0)	2 (100)	0 (0)
Fungal ball	1	0 (0)	0 (0)	0 (0)	0 (0)

*Abbreviations*: EBUS‐GS, endobronchial ultrasound using a guide sheath; NOS, not otherwise specified; NSCLC, non‐small cell lung cancer.

Of the benign lesions (*n* = 43), 37 (86.0%) were diagnosed successfully by RP‐EBUS‐TBLB. Of these, nontuberculous mycobacterial lesions (*n* = 15, 34.9%) were the most common, followed by tuberculosis (*n* = 14, 32.6%) and lung abscesses (*n* = 14, 23.3%). For the 43 benign lesions, one (2.3%) brushing cytology and 24 (55.8%) histology tests were diagnostic, as were 24 (55.8%) microbiology tests. The diagnostic accuracies of RP‐EBUS‐TBLB used to evaluate cavitary PPLs in terms of diagnosing pulmonary tuberculosis and nontuberculous mycobacterial lesions were 85.7% and 80.0%, respectively. All lung abscesses, aspergillosis cases, and inflammatory lesions were successfully diagnosed histologically. The false negatives (*n* = 6) were diagnosed on the basis of sputum microbiology results (*n* = 5), and clinical judgment was informed by radiological monitoring (*n* = 1).

The diagnostic performance of RP‐EBUS‐TBLB in terms of cavitary PPLs is summarized in Table 3. The overall sensitivity, specificity, positive predictive value, negative predictive value, and diagnostic accuracy of RP‐EBUS‐TBLB were 72.5% (29/40), 100% (37/37), 100% (29/29), 77.1% (37/48), and 85.7% (66/77), respectively.

**TABLE 3:** Diagnostic performance of RP‐EBUS‐TBLB used to evaluate cavitary PPLs

Parameter	Final diagnostic conclusions (*n* = 77)
True‐positive, *n*	29
True‐negative, *n*	37
False‐positive, *n*	0
False‐negative, *n*	11
Sensitivity, %	72.5
Specificity, %	100.0
PPV, %	100.0
NPV, %	77.1
Diagnostic accuracy	85.7

*Abbreviations*: NPV, negative predictive value; PPLs, peripheral pulmonary lesions; PPV, positive predictive value; RP‐EBUS‐TBLB, radial probe endobronchial ultrasound‐guided transbronchial lung biopsy.

### Factors affecting diagnostic success

**FIGURE 3:**
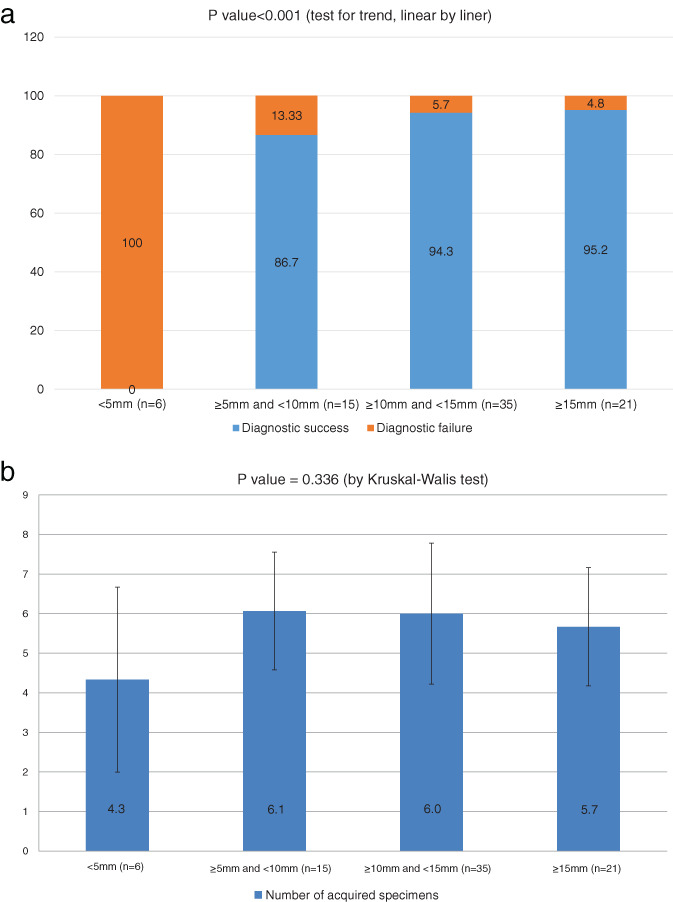
(a) Diagnostic accuracy by cavity wall thickness. (b) Number of specimens by cavity wall thickness

We sought factors affecting PPL diagnostic success (Table 4). Univariate analyses revealed that a thicker cavity wall (≥10 mm, odds ratio [OR] 10.87, 95% confidence interval [CI] 2.53–46.77, *p* = 0.001), a bronchus sign evident in chest CT (OR 6.98, 95% CI 1.51–32.19, *p* = 0.020), and an EBUS image within the lesion (OR 8.33, 95% CI 1.95–35.65, *p* = 0.007) were significantly associated with diagnostic success. The cavity PPL size did not differ between the two groups (OR 0.98, 95% CI 0.94–1.02, *p* = 0.355). Multivariate analyses revealed that a thicker cavity wall (≥10 mm, OR 14.22, 95% CI 2.58–78.35, *p* = 0.002) and an EBUS image within the lesion (OR 12.02, 95% CI 1.91–75.53, *p* = 0.008) independently enhanced diagnostic success. The probability of success increased with increasing thickness of the cavity wall (*p* < 0.001, test for trend) (Figure 3(a)). Diagnosis failed in all patients with cavity wall thicknesses <5 mm. Six patients with cavity wall thicknesses <5 mm were finally diagnosed as lung cancer (two patients), pulmonary tuberculosis (one patient), nontuberculous mycobacteria (two patients), and fungal ball (one patient). The diagnostic success rate was 86.7% in patients with cavity wall thicknesses ≥5 mm and <10 mm. In patients with cavity wall thicknesses ≥15 mm, the diagnostic success rate was 95.2%. However, the number of acquired specimens did not significantly differ by cavity wall thickness (Figure 3(b)).

**TABLE 4:** Factors affecting the diagnostic success of RP‐EBUS‐TBLB in patients with cavitary PPLs

	Diagnostic success (*n* = 66)	Diagnostic failure (*n* = 11)	Univariate analyses		Multivariate analyses	
			Odds ratio (95% confidence interval)	*p* value	Odds ratio (95% confidence interval)	*p* value
Age, years	68.1 ± 11.2	67.1 ± 16.1	0.99 (0.94–1.05)	0.790		
Male	53 (80.3)	6 (54.5)	3.40 (0.90–12.88)	0.116		
Diameter, mm	36.8 ± 15.5	33.1 ± 14.6	0.98 (0.94–1.02)	0.355		
Distance from pleura, mm	8.2 ± 9.0	13.0 ± 15.6	1.04 (0.98–1.10)	0.150		
Cavity wall thickness, mm	13.2 ± 4.9	6.7 ± 4.7	1.50 (1.19–1.89)	0.001		
≥ 10	53 (80.3)	3 (27.3)	10.87 (2.53–46.77)	0.001	14.22 (2.58–78.35)	0.002
< 10	13 (19.7)	8 (72.7)			1.00	
Lobar location						
Upper/middle	35 (53.0)	6 (54.5)	1.06 (0.30–3.83)	0.926		
Lower	31 (47.0)	5 (45.5)	1.00			
Bronchus sign evident on chest CT						
Yes	61 (92.4)	7 (63.6)	6.98 (1.51–32.19)	0.020		
No	5 (7.6)	4 (36.4)	1.00			
EBUS image						
Within the lesion	60 (90.9)	6 (54.5)	8.33 (1.95–35.65)	0.007	12.02 (1.91–75.53)	0.008
Adjacent to the lesion	6 (9.1)	5 (45.5)	1.00		1.00	

*Abbreviations*: CT, computed tomography; EBUS, endobronchial ultrasound; PPLs, peripheral pulmonary lesions; RP‐EBUS‐TBLB, radial probe endobronchial ultrasound‐guided transbronchial lung biopsy.

### Molecular study of malignancies confirmed by RP‐EBUS‐TBLB

The proportions of specimens that allowed molecular study in patients diagnosed with malignancies by RP‐EBUS‐TBLB are listed in Table 5. All malignant specimens were analyzed in terms of epidermal growth factor receptor (EGFR) mutation, anaplastic lymphoma kinase (ALK) immunohistochemistry (IHC), reactive oxygen species proto‐oncogene 1 receptor tyrosine kinase (ROS‐1), and programmed death‐ligand 1 (PD‐L1) IHC. The proportions of specimens allowing analyses of EGFR mutation, and ALK, ROS‐1, and PD‐L1 IHC were 100%, 82.8%, 82.8%, and 86.2%, respectively.

**TABLE 5:** Proportions of specimens allowing molecular study of malignancies confirmed via RP‐EBUS‐TBLB (*n* = 29)

Molecular study	All *n* = 29, (%)	Adenocarcinoma *n* = 11, (%)	Squamous cell carcinoma *n* = 14, (%)	NSCLC, NOS *n* = 4, (%)
EGFR	29 (100)	11 (100)	14 (100)	4 (100)
ALK IHC	24 (82.8)	9 (81.8)	13 (92.9)	2 (50)
ROS‐1 RT‐PCR	24 (82.8)	9 (81.8)	13 (92.9)	2 (50)
PD‐L1 IHC	25 (86.2)	9 (81.8)	14 (100)	2 (50)

*Abbreviations*: ALK, anaplastic lymphoma kinase; EBUS‐GS, endobronchial ultrasound with a guide sheath; EGFR, epidermal growth factor receptor; IHC, immunohistochemistry; NOS, not otherwise specified; NSCLC, non‐small cell lung cancer; PD‐L1, programmed death‐ligand 1; ROS‐1, reactive oxygen species proto‐oncogene 1 receptor tyrosine kinase; RP‐EBUS‐TBLB, radial probe endobronchial ultrasound‐guided transbronchial lung biopsy; RT‐PCR, real‐time polymerase chain reaction.

### Complications

We encountered four cases of infection (5.2%) and three of pneumothorax (3.9%) in the 77 patients who underwent RP‐EBUS‐TBLB. Among the four cases of infection, three recovered with antibiotics and one developed empyema requiring chest tube insertion. All three patients with pneumothorax recovered following oxygen therapy.

## DISCUSSION

We found that that RP‐EBUS‐TBLB was very accurate and safe when used to diagnose cavity PPLs. The diagnostic accuracy was 85.7% and the complication rate was 9.1% (infection 5.2% and pneumothorax 3.9%). Analyses of brushing cytology and microbiology samples, in addition to histology samples, increased the diagnostic rates of both malignant and benign lesions. A thicker cavity wall (≥10 mm) and an EBUS image within the lesion independently enhanced success. The probability of success increased with increasing thickness of the cavity wall, and all obtained specimens allowed molecular evaluation. To the best of our knowledge, this is the largest study to date that has analyzed the utility of RP‐EBUS‐TBLB in terms of diagnosing cavitary PPLs. Furthermore, this is the first study to reveal the positive relationship between cavity wall thickness and diagnostic rate in the diagnosis of cavitary PPLs.

A pulmonary cavity is a gas‐filled space within a zone of pulmonary consolidation, a mass, or a nodule. Cavities in patients with benign lung disease may be caused by necrosis (suppurative, caseous, or ischemic), cystic dilation of a lung structure, or displacement of lung tissue by a cystic structure. Cavities in patients with malignant lung disease may be caused by treatment‐related necrosis, internal cyst formation, or internal desquamation of tumor cells with subsequent liquefaction.^1^ Radiologically, a notch and an irregular internal wall were more common in malignant cavitary lesions; a linear margin, satellite nodules surrounding the cavity, bronchial wall thickening, consolidation, and ground‐glass attenuation were more frequent in benign cavitary lesions.^9^ However, biopsy is often required to confirm diagnosis because the chest CT findings of benign and malignant cavitary PPLs overlap.

Prior to the introduction of RP‐EBUS, TTNB was commonly used to diagnose cavitary PPLs. In a previous study, CT‐guided fine needle aspiration biopsy of cavitary pulmonary lesions was associated with a high diagnostic accuracy (96.1%).^6^ However, the complications included pneumothorax (8.8%), alveolar hemorrhage (13.7%), and hemoptysis (1%). Although fine needle aspiration biopsy (rather than core needle biopsy using cutting needles) was employed, the complication rates were relatively high. In another study, the overall accuracy of TTNB in terms of diagnosing cavitary lesions was 81%.^5^ The overall complication rate was 28%, with pneumothorax being the most common problem (24.5%); 9.4% of patients required chest tube insertion, 1.9% developed mild hemoptysis, and 1.9% had a small hemothorax. In summary, TTNB used to diagnose cavitary PPLs affords a relatively high diagnostic rate (>80%), but the procedure‐related complication rate is also high (≥20%).

Only one prior study has analyzed the utility of RP‐EBUS in terms of cavitary PPL diagnosis (50 lesions).^10^ In that study, the overall diagnostic accuracy was 80%, 77.8% for malignant and 82.6% for benign lesions. An EBUS probe within the lesion was the only factor that enhanced diagnostic accuracy. Of the 50 procedures, only one pulmonary infection occurred. No other major complication, such as pneumothorax or significant hemorrhage, was reported.

In this study, we analyzed 77 cavitary PPLs. In agreement with the previous study,^10^ we found that localization of the probe within the lesion independently enhanced diagnostic success. However, our most interesting result is that a thicker cavity wall (≥10 mm), not the size of the cavity per se, increased the diagnostic success rate, which increased significantly as the cavity wall thickness increased. In Zhuang et al., the diagnostic accuracy was not associated with cavity wall thickness, but more nondiagnostic samples occured in wall thicknesses <5 mm than ≥5 mm lesions in fine needle aspiration biopsies of cavitary PPLs.^6^ Kiranantawat et al. reported that wall thickness at the biopsy site is a significantly independent factor in terms of diagnostic success.^5^ Although the number of acquired specimens did not differ significantly by cavity wall thickness in the present study, we recommend targeting the thickest wall when guiding RP‐EBUS for TBLB; this optimizes tissue samples. In our experience, cavitary walls of thickness <5 mm tend to yield unsatisfactory samples. Although the reason for this is unclear, not using fluoroscopy may have affected these results. Further study is needed to determine the relationship between cavitary wall thickness and tissue sample quality.

As in the previous study,^10^ we found that analyses of brushing cytology and microbiology samples (in addition to histology samples) increased the diagnostic rate. The final histology diagnosis rates for malignant and benign lesions were 79.4% and 55.8%, respectively. However, after adding brushing cytology and microbiology data, these figures increased to 85.3% and 86.0%. In particular, microbiology tests were very valuable in terms of diagnosing benign lung disease. After biopsy and GS removal, efforts must be made to obtain enough bronchial washing samples for microbiology tests with the bronchoscope wedging as close to the lesion as possible.

Only a few studies have explored the feasibility of molecular studies of lung cancer confirmed via RP‐EBUS. One study reported that specimens obtained via RP‐EBUS‐TBLB using a 1.8‐mm diameter biopsy forceps allowed analyses of EGFR mutational status, and ALK and PD‐L1 IHC, in more than 90% of adenocarcinoma cases.^11^ One French study revealed that multigene molecular analyses could be performed on almost 80% of specimens sampled via RP‐EBUS‐TBLB using a 1.5‐mm diameter microbiopsy forceps.^12^ We used such forceps, and the statuses of EGFR mutations, ALK, ROS‐1, and PD‐L1 IHC were evaluable in more than 80% of samples, comparable to a previous study.^12^

Our RP‐EBUS‐TBLB complication rates (3.9% for pneumothorax and 5.2% for infection) are higher than those of previous RP‐EBUS‐TBLB studies. One study on 965 PPLs reported an overall complication rate of 1.3%: 0.8% pneumothorax and 0.5% pulmonary infection.^13^ A meta‐analysis revealed that pneumothorax developed in 1.0% of patients, of whom 0.4% required chest tube insertion.^14^ Several complications were possible in the present study, for two reasons. First, in terms of pneumothorax, many patients had underlying lung disease (in particular, 29.9% had COPD). A previous study found that pneumothorax developed in 3.3% of such patients and that pulmonary emphysema was an independent risk factor for pneumothorax.^15^ Second, in terms of infection, a recent study showed that infections developed in 4.47% of patients, and cavitation in the lesion was significantly associated with infectious complications after RP‐EBUS‐TBLB (OR 3.63, 95% CI 1.51–8.71).^16^ Cavities are sometimes caused by necrosis; biopsy itself may exacerbate infection in a necrotic area caused by pathogens transported (via the bronchoscope) from the oral cavity and upper airway. However, our complication rates were much lower than those of studies that biopsied cavitary PPLs using TTNB.^5^, ^6^

Our work had several limitations. First, this was a single‐center retrospective study on a small number of cavitary PPL patients; the results cannot be generalized. Second, we performed biopsy without fluoroscopic guidance to diagnose PPLs and this may have affected the diagnostic accuracy. The use of fluoroscopy help to check whether the forceps are within the lesion and assist TBLB in the lower lobe lesions, increasing the diagnostic accuracy. Third, all procedures were performed by three pulmonologists, who may have differed (to some extent) in competence. However, all had at least 5 years of experience in performing many respiratory procedures and all used standardized protocols. Thus, among‐examiner differences are unlikely to be significant.

## CONCLUSIONS

RP‐EBUS‐TBLB of cavitary PPLs affords a high diagnostic accuracy with an acceptable complication rate. A thicker cavity wall (≥10 mm) and EBUS imaging when the probe lay within the lesion independently enhanced the diagnostic success rates. The success rate increased with increasing thickness of the cavity wall. Most specimens were sufficiently large to allow molecular malignancy confirmation.

## DECLARATIONS

This study was conducted in accordance with the tenets of the Declaration of Helsinki, and the study protocol was reviewed and approved by the Institutional Review Board of Yeungnam University Hospital (YUH IRB no. 2020–09–025). The need for informed patient consent was waived because the study was retrospective in nature.

## CONSENT FOR PUBLICATION

Not applicable.

## CONFLICT OF INTERESTS

No authors have any conflicts of interest to report.
